# Maternal technoference decreases brain-to-brain synchrony during mother-infant interaction

**DOI:** 10.1038/s41598-026-37037-5

**Published:** 2026-01-28

**Authors:** Marion I. van den Heuvel, Agata Mosińska, Elise Turk, Maryam Alimardani

**Affiliations:** 1 Tranzo Scientific Center for Care and Wellbeing, Tilburg University, Tilburg, The Netherlands; 2 Department of Cognitive Science and AI, Tilburg University, Tilburg, The Netherlands; 3 Department of Experimental Psychology, Utrecht University, Utrecht, The Netherlands; 4 UMC Utrecht Brain Center, University Medical Center Utrecht, Utrecht, The Netherlands; 5 Department of Computer Science, Vrije Universiteit Amsterdam, Amsterdam, The Netherlands

**Keywords:** EEG hyperscanning, Mother-infant interaction, Smartphone use, Technoference, Brain-to-brain synchrony, Neuroscience, Psychology, Psychology

## Abstract

Face-to-face interactions between parents and infants are crucial for healthy infant development. In today’s world, these interactions are frequently disrupted by parental distraction by technological devices, a phenomenon known as *technoference*. This study aimed to investigate the effects of technoference on mother-infant brain-to-brain synchrony – a measure for how well two brains are communicating. Previous research has shown that greater brain-to-brain synchrony may reflect higher sensitive caregiving, while lower synchrony reflects higher intrusive caregiving. A total of 33 mother-infant dyads participated in a modified Still-Face Paradigm incorporating maternal smartphone distraction. Dual-EEG was employed to measure mother-infant brain-to-brain synchrony which was subsequently quantified using weighted Phase Lag Index (wPLI). Results revealed that, as expected, mother-infant brain-to-brain synchrony was decreased during the smartphone interruptions. Additionally, brain-to-brain synchrony between mother and infant went back to baseline during reunion. Overall, these findings align with previous research emphasizing the potential disruptive effect of smartphones in parent-infant interactions, but also suggest that mother-infant brain-to-brain synchrony can be restored when the mother re-engages in the interaction.

## Introduction

The widespread adoption of mobile phones, with 93% of the European population aged 10 and above owning one, have brought about a profound shift in social norms and the nature of social interactions^1,2^. One significant aspect of this shift in social dynamics is captured by the concept of technology interference or *technoference* which refers to the interruptions in social interactions caused by digital devices^3,4^. Parental technoference, or sometimes referred to as “parental phubbing”^5^, has received particular scrutiny as portable devices facilitate the use of digital media during family activities, such as feeding/mealtimes, bedtime routines, and play time. These activities are vital for a child’s socio-emotional health^3,6–8^ and recent research indicates that introduction of digital media may disrupt positive interaction during these family activities^9,10^.

As smartphone use continues to rise in parents, a growing body of research pointed to the detrimental effects of parents’ phone usage on their interactions with children. Research has shown that technoference is associated with lower parenting quality^11^ and lower verbal and non-verbal interactions between parents and their children^12^, which in turn can impact language development^13,14^. Smartphone distraction has also been found to lower parental responsiveness and sensitivity to child bids (i.e., child’s attempt to connect or get attention from another person)^15^. Parents’ excessive engagement with their phones can also contribute to children engaging in risky behaviors as they seek attention from their distracted parents, leading to more injuries^16^. Such disruptions in parent-child interactions have far-reaching consequences as they may adversely impact the development of a secure attachment style, which is crucial for a child’s socio-emotional development^6,7^.

The mother-infant relationship is particularly important for child’s socio-emotional development in later life^17^. This relationship is characterized by dyadic interactions that encompass both verbal and nonverbal levels^18^, as well as their biological systems^19^, resulting in synchrony not only in their behaviors but also in their physiological processes^19,20^. Biobehavioral synchrony forms the foundation for the healthy development of mother-infant bonds^7,21^. Synchronized mother-infant behavior experienced by the infant during this sensitive period has been found to positively predict infant and child self-regulation, theory of mind in childhood, and healthy brain development^22–24^.

Advancements in neuroimaging techniques have provided developmental researchers with new opportunities to broaden the concept of biobehavioral synchrony in parent-infant dyads^25^. *Hyperscanning* enables the simultaneous measurement of brain activity from multiple individuals, facilitating a deeper understanding of the neural dynamics underlying social interactions^26–28^. This technique has emerged as a valuable tool in developmental research, enabling the measurement of brain-to-brain synchrony between parents and infants, utilizing a range of neuroimaging techniques, such as dual-fNIRS, dual-MEG, and dual-EEG^25,29–32^. Dual-EEG is particularly suitable for measuring brain activity in mother-infant dyads due to its non-invasive and infant-friendly nature^32^. Additionally, the excellent temporal resolution of EEG can “keep up” with the fast dynamics of social interaction.

Still, the body of literature on dual-EEG studies involving mothers and infants is very limited. Initial findings have shed light on the nature of the mother-infant bond. For instance, it has been shown that infants experience greater brain-to-brain synchrony during positive interactions with their mothers^33,34^, while experiencing lower synchrony during maternal intrusive interaction^33^. Research also showed that brain-to-brain synchrony between mother and infant increased during affective touch and proximity^35^ and when maternal body odor cues were presented to the infant^36^. Together, these results confirm that mother-infant synchrony seems to reflect interaction quality and the underlying relationship quality between mother and infant, however, no previous research has investigated the impact of maternal distractions caused by technoference on mother-infant synchrony at the neurophysiological level.

While there is behavioral evidence for the negative impact of digital devices on the parent-infant interaction quality (for a review, see^3,37^, the neurophysiological evidence is limited. The studies that do exist mainly focus on older children. For instance, Zivan, et al.^38^ studied technoference in mother-toddler dyads. The authors investigated synchronization between the brains of 24 mothers and toddlers (mean age = 33.5 months ± 5.8 months) during a dialogic storytelling (reading) task that was either interrupted by maternal smartphone use or uninterrupted. The results indicated that interruptions during the task reduced brain-to-brain synchrony between the mother’s language-related regions and the child’s comprehension-related regions in the alpha frequency band, compared to the uninterrupted condition. These findings support previous behavioral data and provide neurophysiological evidence for the negative impact of mobile phone use on parent-child interactions in toddlerhood. Yet, research investigating the impact of maternal distractions caused by technoference on mother-child brain-to-brain synchrony in infancy is still lacking. This is an important gap, since infancy is a period of extreme sensitivity to experiences that promote (or hinder) development, and in which the benefits of early childhood interventions are amplified^39^.

**Fig. 1:**
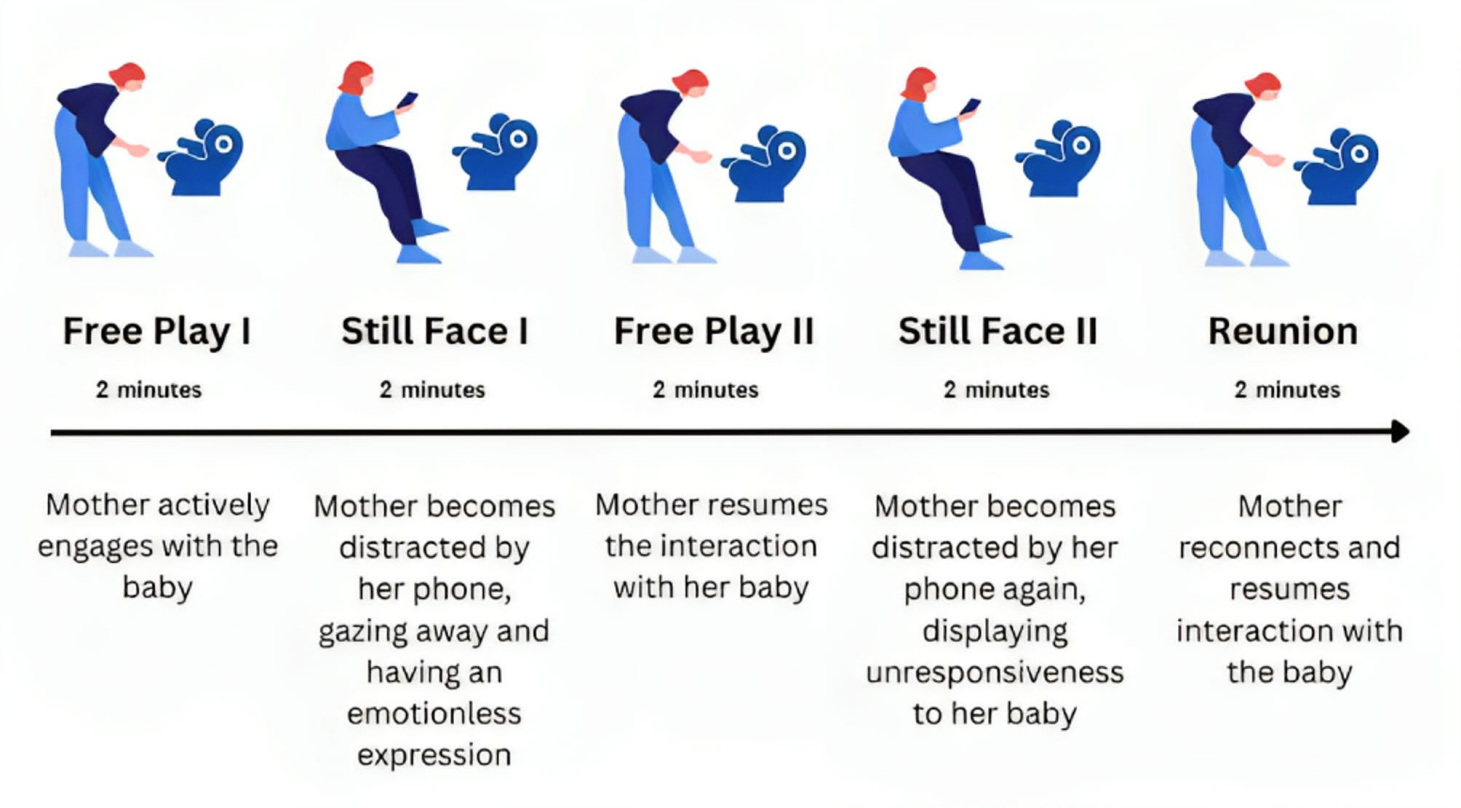
Schematic representation of the adapted still-face paradigm procedure.

The present study sought to bridge this gap by investigating the effects of maternal smartphone distraction on mother-infant brain-to-brain synchrony. To assess the level of synchrony, mother-infant dual-EEG was recorded during interaction within a modified Still-Face Paradigm (SFP; Tronick et al., 1978). The SFP consists of a mother-infant interaction which is interrupted two times by a period of (emotional) unavailability of the mother, or the so-called “still-face”. Originally, the mother would be unresponsive, with a blank expression during this period^40^. Here, we adapted this period by asking the mother to look at her smartphone and be unresponsive cf^41,42^. After each “still-face” period, the mother is asked to reengage/reunite with her infant (reunion phase). More specifically, the “smartphone-adapted Still-Face Paradigm (SFP)” that was used in the current study consisted of five phases of each 2 min: (1) first free play (FP1), (2) first Still-Face (SF1), (3) second free play (FP2), (4) second Still-Face (SF2), (5) reunion (RU; free play) (see Fig. 1). A graphical overview of the smartphone-adapted Still-Face Paradigm is presented in Fig. 1. In line with previous EEG studies on mother-infant brain-to-brain synchrony^33,36^, the weighted Phase Lag Index (wPLI) was used as a measure of brain-to-brain synchrony^43,44^. The current study focused on the infant theta (3–5 Hz) and alpha (6–9 Hz) frequency bands, as these bands have been found to play a significant role in social interactions^25,33,45^.

We expected that brain-to-brain synchrony would decrease during the still-face periods, when the mother was unresponsive to the infant and ‘absorbed’ by her smartphone. Additionally, we explored whether brain-to-brain synchrony would recover to baseline level in the reunion phases, in which the mother tried to reconnect. Based on previous research, it is currently unclear whether brain-to-brain synchrony would recover or not after smartphone-interruption. While several studies show that infant behaviors (e.g., room exploration, toy engagement) and infant mood (positive and negative affect) often do not return to baseline after the still-face period e.g.,^41,42,46,47^, other studies have shown that, in contrast, the physiological responses, such as the cardiovascular system, do return to baseline e.g.,^48,49^. The script that was used for the analyses is publicly available and can be retrieved from Github: https://github.com/m-agat/mother_infant_neural_synchrony.

## Results

### Descriptive statistics

The final sample consisted of 33 mother-infant dyads, with a mean infant age of 8.9 months (range = 5–12 months; SD = 1.3). There were 15 male infants and 18 female infants included. Mothers had a mean age of 32.9 years (range = 27–42 years; SD = 3.4) and were predominantly of Dutch nationality, except for 1 German, 1 Hungarian, and 1 Polish mother. All mothers had a Western European ethnicity, except one mother who had a Surinam background. Almost 70% (*n* = 23) of mothers indicated that they divided the care for their infant equally with their partner, compared to the other 30% (*n* = 10) of mothers who indicated to be the main caregiver. The mothers were relatively highly educated, with over 50% having a university degree. According to the Dutch version of the Postpartum Bonding Questionnaire^50^ that mothers filled out after the visit, none of the mothers scored above the cut-off for bonding issues with their infant, indicating normal bonding in all included dyads. Sample characteristics are presented in Table 1.

**Table 1:** Sample characteristics.

Participant	*N*	M (SD)	Min	Max
Mothers	33			
Age at EEG measurement (years)		32 (6.25)	27	42
Education				
MBO (middle-level applied education)	3			
HBO (higher professional education)	9			
University	15			
Higher academic (PhD)	6			
Infants	33			
Age at EEG measurement (months)		9.29 (1.38)	5.72	12.16
Sex				
Girl	18			
Boy	15			

###  Sex and age differences in brain-to-brain synchrony

We first explored sex and age differences in brain-to-brain synchrony as quantified by wPLI within our sample. We found a significant difference in mother-infant brain-to-brain synchrony between dyads with a male infant versus dyads with a female infant. Specifically, we found that mother-infant brain-to-brain synchrony in the alpha band was slightly higher in mother-daughter dyads (*M* = 0.37) during the second Still-Face condition (*t*(26.77) = −2.129, *p* = 0.043) as compared to mother-son dyads (*M* = 0.36). Additionally, we explored age differences in brain-to-brain synchrony measures. Here, we found that dyads with an older infant showed significantly lower brain-to-brain synchrony in the first Still-Face condition, as measured in the theta band (*r* = −0.370, *p* = 0.034). Therefore, for the main analyses, infant sex and age were included as covariates into the model.

### Brain-to-brain synchrony in infant theta band (3–5 Hz)

First, a validation process was conducted, in which brain-to-brain synchrony in each epoch was compared to brain-to-brain synchrony in a surrogate dataset including shuffled EEG signals from the mother and infant. This was done to differentiate real synchrony from background fluctuations. If the observed wPLI was higher than 95% of the randomized wPLIs, it was concluded that the observed synchrony was not due to random noise but rather due to the true synchronization between the EEG of the mother and infant. The trials in which wPLIs were found insignificant were not included in the computations of the global inter-brain synchrony. For the wPLI in the theta band, it was found that over 95% of the 2-second epochs in each condition could be included. Based on these results, we concluded that wPLI is a robust measure of brain-to-brain synchrony and can be used for the purpose of this study. Next, we explored the dataset for outliers. No outliers were detected for the theta band wPLI values. A summary of the mean values of brain-to-brain synchrony per condition and standard deviations can be found in Table 2.

**Table 2:** Summary of brain-to-brain synchrony values across conditions for alpha and theta band.

	Free play 1	Still face 1	Free play 2	Still face 2	Reunion
Alpha band, mean (SD)	0.364 (0.017)	0.357 (0.017)	0.367 (0.018)	0.358 (0.019)	0.373 (0.021)
Theta band, mean (SD)	0.412 (0.032)	0.399 (0.023)	0.415 (0.028)	0.415 (0.035)	0.420 (0.023)

The results of the Friedman test for the theta band revealed a statistically significant overall difference between conditions, *χ²*(df = 4) = 19.078, *p* < 0.001, indicating a significant variation in mother-infant wPLIs between conditions. The covariates infant sex and age did not show any significant main or interaction effects.

**Fig. 2:**
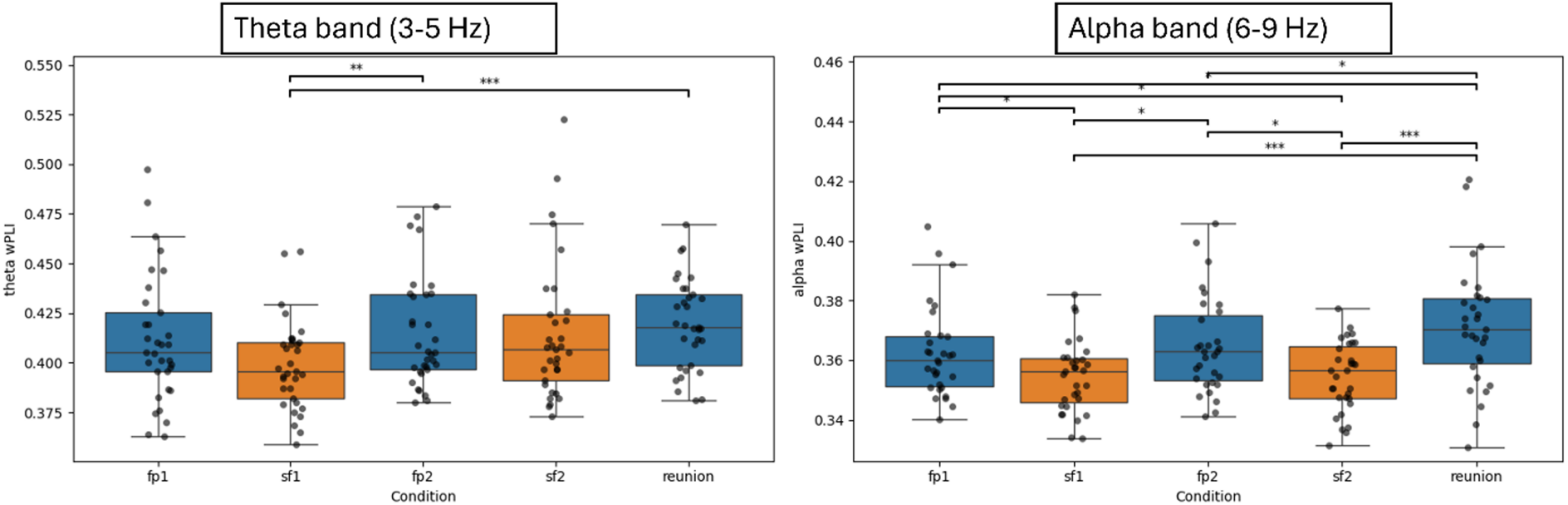
Graphical overview of the global weighted PLI (wPLI) values over the course of the adapted still-face Paradigm in the theta band (panel **A**) and the alpha band (panel **B**). Statistically significant differences were indicated by an asterix (*). Non-parametric tests were used (pairwise wilcoxon signed-rank tests) and all p-values were FDR-corrected.

Following the Friedman test, post hoc comparisons were conducted with Pairwise Wilcoxon signed-rank tests and FDR correction for multiple testing. The results for the theta band revealed significant differences between conditions, showing a pattern of decreases in brain-to-brain synchrony mostly during the first Still-Face condition and a return to baseline in brain-to-brain synchrony during the Reunion Condition. Specifically, the Still-Face 1 (SF1) condition showed a significantly lower brain-to-brain synchrony compared to Free Play 1 (FP1) condition (*W* = 94.0, *p* = 0.003, *FDR-corrected*) and the Reunion (RU) (*W* = 68.0, *p* < 0.001). SF2, however, showed no significant differences with the other conditions. For a graphical overview of the results in the theta band, see Fig. 2A.

### Brain-to-brain synchrony in infant alpha band (6–9 Hz)

For the wPLI in the alpha band, we again conducted the comparison of the recorded data with surrogate randomized signals and found that over 95% of the 2-second epochs in each condition could be included. Here, 2 outliers were detected and removed from the data: one wPLI value in the SF1 condition and one wPLI value in the SF2 condition (different dyads). A summary of the mean values of brain-to-brain synchrony per condition and standard deviations in the theta band can be found in Table 2.

Similar to the theta band, the results of the Friedman test for the alpha band revealed a statistically significant difference in mother-infant synchrony over the course of the still-face paradigm *χ²*(df = 4) = 25.9152, *p* < 0.001. This finding indicates a significant variation in mother-infant wPLIs between conditions. Again, the covariates infant sex and age did not show any significant main or interaction effects.

Following the Friedman test, post hoc comparisons were conducted using Pairwise Wilcoxon signed-rank tests with FDR correction for multiple testing. Similar to the results of the theta band, the analysis revealed several significant differences between phases. Overall, the results revealed a pattern of decreases in brain-to-brain synchrony during both Still-Face conditions and an increase in brain-to-brain synchrony during the Reunion condition. Specifically, brain-to-brain synchrony in Still-Face 1 (SF1) condition was significantly lower than the both Free Play conditions (FP1: *W* = 133.0, *p* = 0.034, *FDR-corrected*; FP2: *W* = 140.0, *p* = 0.028, *FDR-corrected*) and the Reunion condition (RU: *W* = 76.0, *p* = 0.001, *FDR-corrected*). Similarly, brain-to-brain synchrony in Still-Face 2 (SF2) was significantly lower than both the Free Play conditions (FP1: *W* = 135, *p* = 0.027, *FDR-corrected*; FP2: *W* = 135, *p* = 0.027, *FDR-corrected*) and the Reunion condition (RU: W = 83.0, *p* = 0.001, *FDR-corrected*). Additionally, a significant difference was observed between both Free Play conditions and RU conditions (FP1: *W* = 145.0, *p* = 0.029, *FDR-corrected*; FP2: *W* = 136.0, *p* = 0.034, *FDR-corrected*). For a graphical overview of the results in the alpha band, see Fig. 2B.

### Topographical analyses of inter-brain regions

We then continued our analyses on a regional level to examine what inter-brain regions showed a still-face effect. To explore this, we compared mother-infant inter-brain regions between the Still-Face conditions (SF1 and SF2) and the Free Play and Reunion (FR1, FP2, RU) conditions for theta and alpha bands separately.

In the alpha band, significant decreases in brain-to-brain synchrony were observed between several regions of the baby and the mother during the Still-Face (SF1, SF2) conditions compared to the Free Play and reunion (FP1, FP2, RU) conditions. Specifically, synchrony between the baby’s frontal region and the mother’s occipital region (*t* = −1.79, *p* = 0.039), as well as between the baby’s central region and the mother’s right temporal region (*t* = −1.70, *p* = 0.047), significantly decreased during the Still-Face condition. Additionally, reductions in synchrony were found between the baby’s occipital region and the mother’s frontal (*t* = −2.01, *p* = 0.024), central (*t* = −2.39, *p* = 0.010), left temporal (*t* = −2.34, *p* = 0.011), and right temporal (*t* = −2.88, *p* = 0.003) regions.

In the theta band, brain-to-brain synchrony showed significant reductions during the Still-Face (SF1, SF2) conditions compared to the Free Play and Reunion (FP1, FP2, RU) conditions. Notably, synchrony between the baby’s left temporal region and the mother’s occipital region was significantly reduced (*t* = −2.11, *p* = 0.020). Further decreases were observed between the baby’s occipital region and the mother’s frontal (*t* = −1.98, *p* = 0.026) and central-parietal regions (*t* = −1.82, *p* = 0.037).

**Fig. 3:**
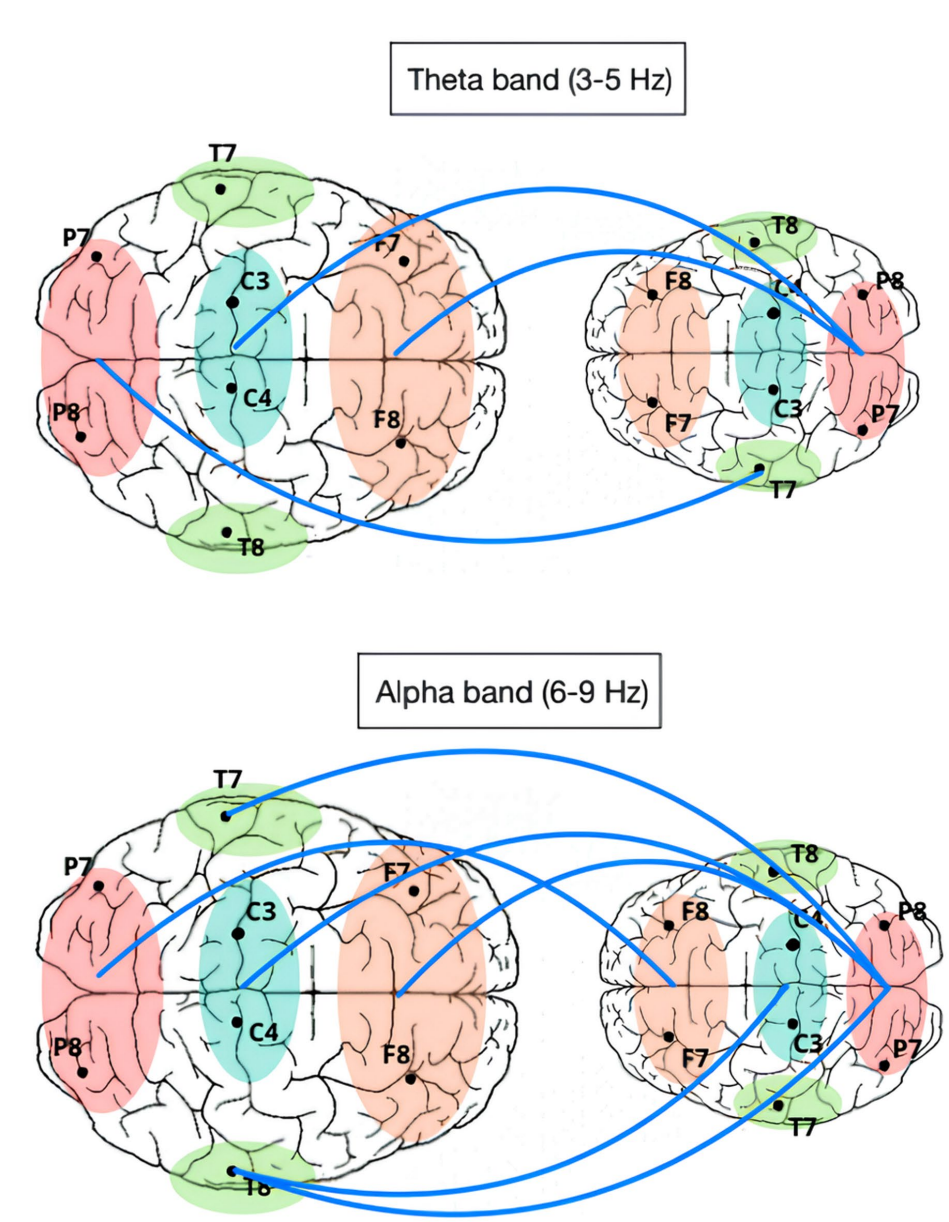
Graphical overview of inter-brain regions that showed significant *lower* brain-to-brain synchrony in theta band (upper panel) and alpha band (lower panel) during the Still-Face conditions (SF1 and SF2) as compared to the Free Play and Reunion (FR1, FP2, RU) conditions of the adapted Still-Face Paradigm. Displayed are axial views of the parent (left) and infant (right) brains. Frontal regions are shown in orange, temporal regions in green, occipital regions in red and central-parietal regions in blue.

In general, the results showed that lower synchrony between the maternal and infant brain during maternal technoference was widespread throughout the brain, involving temporal, parietal, occipital and frontal areas. Overall, we observed stronger and more widespread effects in the alpha band. For a graphical overview of the inter-brain connections that were significantly different between conditions, see Fig. 3.

### Sensitivity analyses

To check the robustness of our findings, we conducted several post hoc sensitivity analyses. First, we reran the analyses with parametric testing, i.e., repeated-measures ANCOVA. The parametric tests revealed a similar pattern of results, but with more significant findings for the theta band (i.e., SF1 < FP1 and SF1 < SF2). Next, we performed drop-out analyses to compare dyads that were included in the final sample (*N* = 33) versus dyads of which the EEG data could not be included (*N* = 9; either because the data were too noisy or because the task was not performed or stopped). There were no significant differences between the two groups regarding the age of the infant, the age of the mother, or the temperament of the infant (all p-values > 0.05). These results indicate that infants were not excluded due to factors that could bias our results.

## Discussion

The primary objective of the present study was to investigate how maternal unavailability caused by smartphone distraction during social interaction affects mother-infant brain-to-brain synchrony. In line with our expectations, we observed a clear pattern of decreased brain-to-brain synchrony between mother and infant in periods of smartphone interference, in which the mother was distracted by her phone and asked to be unresponsive. Additionally, our results showed that brain-to-brain synchrony recovered, and sometimes even increased, when the interaction was resumed after smartphone distraction. Topographical analyses further showed that the effect of smartphone interruptions on brain-to-brain synchrony was widespread throughout the brain, involving central, parietal, occipital and frontal areas in both mother and infant. While our results underscore the negative impact of unavailability of the mother due to technoference on mother-infant interactions in the first year of life, it also demonstrates the ability of the brains of mother and infant to reconnect after disturbance.

The findings of our study support the hypothesis that maternal technoference would negatively impact mother-infant brain-to-brain synchrony. These findings correspond with previous behavioral research that showed lower behavioral synchrony and parenting quality during smartphone distraction^4,10^. We found lower brain-to-brain synchrony in the alpha band (6–9 Hz) in response to maternal smartphone distraction, consistent with Zivan, et al.^38^, who reported lower brain-to-brain synchrony in the alpha band (8–12 Hz) in older mother-toddler dyads (24–42 months old) when mothers were distracted by their smartphones during joint storytelling as compared to uninterrupted storytelling. While Zivan, et al.^38^ only examined the alpha band, we explored both (infant) alpha and theta band and found decreases during maternal distraction in both frequency bands, expanding the literature. It should be noted that in the theta band, we did observe a still-face effect for the first still-face episode (SF1) but not for the second still-face episode (SF2). Further research with larger samples should examine whether this lack of significant difference for the second still face in the theta band was due to low power or a genuine effect. Additionally, we showed that decreased brain-to-brain synchrony in response to technoference can already be observed in infancy.

Importantly, our results also show that disturbed brain-to-brain synchrony between mother and infant recovers when the mother reengages in interaction. This result is in line with previous studies on the physiological response, such as respiratory sinus arrhythmia (RSA), of infants during the Still-Face Paradigm. These studies showed that infants generally return to baseline during the reunion phase e.g.,^48,49^. Interestingly, behavioral studies show the opposite – infant behaviors (e.g., room exploration, toy engagement) and infant mood (positive and negative affect) often do not return to baseline during reunion e.g.,^41,42,46,47^. This includes results from the current sample^47^. Together, these results may suggest that mother-infant brain-to-brain synchrony may proceed behavioral reconnection after disconnection. This is an interesting proposal that needs further exploration in multimodal interpersonal synchrony studies^51^. In the alpha band, we even observed (significantly) higher synchronization in the reunion phase as compared to baseline (FP1) and the second free play (FP2). The increased brain-to-brain synchrony could reflect the effort of the mother (and infant) to restore the disconnection and get back on the same wavelength, literally. Our current data, however, does not permit testing this hypothesis.

Decreased synchrony during periods of maternal technoference may be the result of disturbed behavioral interaction cues, such as mutual eye contact, eye gaze, and touch^28,52,53^. The infant often withdrawals from the interaction during the still-face condition, for instance by averting their gaze^54^, which may also lower brain-to-brain synchrony. Yet, there may be alternative explanations for the lower brain-to-brain synchrony during the still-face conditions. An important alternative explanation is the potential entrainment of the brains of mother and infant to the mother’s speech. From previous research, it is known that the brain can track the amplitude envelope of naturalistic speech at multiple rates^55^. Recent research has shown that the infant brain, like the adult brain, is capable of cortical tracking of speech e.g.,^28,56–58^. Crucially, the prosodic stress (around 2 Hz) and syllable (around 5 Hz) frequencies of infant-directed speech fall within the theta band^59^, suggesting the possibility that brain-to-brain synchrony in the theta band may in fact be entrainment of the brains of mother and infant to the frequency of mother’s speech. Since there is no speech in the still-face condition (versus speech in the other conditions), this may have caused lower brain-to-brain synchrony during this condition. Still, we also found robust results in the alpha band, which is less sensitive to neural tracking of sound. Taken together, the decreased brain-to-brain synchrony in periods of smartphone interference is likely the result of a combination of changes in interpersonal communication of the mother while being distracted, including less speech, touch, mutual eye contact and eye gaze and the response of the infant to the technoference, including averted eye gaze.

Another important explanation for our findings needs to be discussed as well. It could be that the decreased brain-to-brain synchrony is not the result of the technoference perse, but of the disconnection with and the unavailability of the mother as a result of the technoference. A still-face effect may also be present when mothers attend to another task, such as book reading. While smartphones can be especially distracting, since they are basically made to keep one’s attention, with the current paradigm we cannot be sure whether the results are due to the use of a smartphone or more generally to a lack of direct interaction. Future research should explore this, by adding additional conditions in which the mother attends to something else, such as a book or a conversation with another person entering the room for two minutes. Previous behavioral research did show that interaction quality deteriorated when parents were distracted, independently of what type of distraction (digital or non-digital)^60^.

The smartphone-adapted Still-Face Paradigm that was used in this study is different from the original (double) Still-Face Paradigm (SFP) in several ways. The most prominent difference is the use of a smartphone during the still-face phases. In the original SFP mothers are not given any task other than not responding to their child and keeping a ‘neutral’ face, in our paradigm mothers were instructed to be ‘absorbed’ in their smartphone and not respond to their child. While they were not instructed to keep a ‘neutral’ face, many mothers had very low expression. Having the smartphone there may also have introduced an additional difference. In the classic SFP, the child has no context as to why the mother stopped responding, in contrast, in the smartphone-adapted SFP, the child can observe the mother attending to another task. Another difference that we introduced to make the task more ecologically valid was that infants could keep their toys. In the classic SFP, the toys are taken away from the child during the still-face episode(s). We noticed that most infants did not engage with their toys during the still-face, due to their distress. The fact that we see a still-face effect in both versions of the task^47^ may suggest that it is the unresponsiveness of the mother that triggers the negative response of the infant, not the unnatural situation (the mother just staring with a neutral face and the infant not having any toys).The current work has several limitations that should be acknowledged. First, the sample size of our study is relatively small. Nevertheless, due to the within-dyad comparison design of our study, this limitation is largely overcome. A post-hoc power analysis showed that, with our current sample size, we have sufficient power to detect large and medium effects (d = 0.8 and d = 0.5, respectively) but insufficient power to detect small effects (d = 0.2). Additionally, the overall quality of the infant and maternal EEG data was high and the number of artefacts low. On average, we were able to keep around one-hundred 2-sec hyper-epochs per participant, corresponding to over 2 min of clean mother-infant EEG data. Second, infant EEG studies, and naturalistic set-ups such as dual-EEG studies with infants in particular, have a high amount of movement artefacts^61^, with the current study being no exception. Currently, no standardized techniques exist to successfully remove movement artefacts from infant EEG data, mostly because artefacts in infant data, such as eye blinks and limb movement, are less stereotypical and therefore harder to identify by algorithms^61^. While our artifact rejection procedure removed a good portion of the (larger) movement artefacts in the infant data, it is still possible that undetected artefacts contaminated our dataset. Third, our sample is homogeneous in demographic, with a high percentage of highly educated, white women and their infants. This may have biased the results, since previous research has reported that demographic differences such as socio-economic status or cultural background can affect the outcome of the Still-Face Paradigm^62,63^. Furthermore, we noticed that not all mothers adhered to the instructions of the Still-Face Paradigm (i.e., not responding to their infant during the still-face condition) and, for instance, touched the infant briefly or returned a toy that fell on the ground. While we instructed the mothers to have no response at all, their behaviors may have made our paradigm more ecologically valid. Moreover, recent research found that breaches of the Still-Face Paradigm instructions are very common – over half of the mothers breached instructions^64^. Importantly, these breaches were unrelated to the infant’s response to the still-face condition^64^, indicating that non-adherence of mothers during the Still-Face Paradigm does not affect the effect of the still-face condition. Finally, future research should consider taking into account the level of exposure of the infant to maternal smartphone use in daily life. Infants with high exposure of parental technoference may respond differently to the smartphone-adapted Still-Face Paradigm than infants with low exposure. Additionally, infant’s own experience with screens may have also affected their response to maternal smartphone use. Future research could include maternal report of own and infant’s screen usage.

The findings of the present study contribute to developmental research investigating the effects of modern technology on human development and of disconnection between mother and infant in general. There seems to be sufficient research to speculate that the observed lower brain-to-brain synchrony during maternal smartphone use reflects lower responsiveness and decreased sensitivity^33,53,65,66^. However, our current limited knowledge of brain-to-brain synchrony in the developing brain and the lack of a theoretical basis for what brain-to-brain synchrony exactly measures, hinders clear interpretation of our results. Still, our findings add to the growing literature underscoring the potential disconnect parental smartphone use can create between parent and infant. Importantly, we also showed that the disconnect, at least at the neural level, can be easily restored when parents reconnect with their infant. Taken together, the current study can be a precursor for future studies to further investigate the impact of screen-based technology on mother-infant brain-to-brain synchrony. Such research can help parents and professionals gain a deeper understanding of how to use digital devices in a mindful and positive manner, and minimize their negative impact on child development.

In sum, the current study showed lower brain-to-brain synchrony in mother-infant dyads when the mother was distracted by her smartphone as compared to when they interacted. Additionally, we demonstrated that mother-infant brain-to-brain synchrony recovered when the mother put her smartphone away and reconnected with her infant. While our results underscore a potential negative impact of technoference (or disconnection in general) on mother-infant interactions in the first year of life, it also demonstrates the ability of the brains of mother and infant to reconnect after disturbance.

## Methods

### Participants

This study was part of the Brains in Sync project (for more information, see OSF project page: https://osf.io/xvy53/). For the purpose of this project, mother and infants were recruited with flyers and via social media. Healthy infants with an age between 5 and 12 months were included. Mothers needed to be over 18 years and able to speak and read Dutch. A total of 41 mother-infant dyads were recruited in this study. For the current study, only dyads with complete EEG data were included. One infant had to be rescheduled several times due to sickness and was over 12 months when they participated. This infant was therefore excluded from the analysis. Additionally, we excluded 4 mother-infant dyads because they did not (fully) complete the interaction task. This was mostly due to excessive crying of the infant or extreme movement (e.g., baby getting out of car seat). After cleaning the data, we had to exclude three more dyads because they did not meet data quality criteria. This resulted in a final sample size of *N* = 33 mother-infant dyads with complete, high-quality data.

### Procedure

The experiment consisted of an online questionnaire that mothers filled out at home and a lab visit at the Life Span Lab of Tilburg University, The Netherlands. The questionnaires included covered a range of topics, including demographics, childcare-related questions, maternal anxiety/depression, mindfulness, and maternal childhood trauma. The questionnaire took about 45–60 min to complete and was conducted using Qualtrics software.

The mother and her infant were then invited to the lab for the dual-EEG measurement. The lab visit, including EEG application and clean-up, took about 1.5 h. Mother and infant were asked to complete several interaction tasks while EEG was being recorded. The following tasks were conducted: a free play interaction (3 min), joint picture book reading (2–5 min), adapted Still-Face Paradigm (10 min), free play interaction (3 min), blowing bubbles (2 min). This study only used data from the adapted Still-Face Paradigm.

After the experiment, the mothers received a small gift for the infant to compensate for their time and effort. All mothers signed written informed consent for participation in the study with their infant. The Ethics Review Board of the School of Social and Behavioral Sciences at Tilburg University, The Netherlands approved the experiment (number: RP34). The study was conducted according to the Declaration of Helsinki.

### The smartphone-adapted still-face paradigm

The smartphone-adapted Still-Face Paradigm (SFP) was based on the double SFP^40,67^ and consisted of five phases of each 2 min: (1) first free play (FP1), (2) first Still-Face (SF1), (3) second free play (FP2), (4) second Still-Face (SF2), (5) reunion (RU; free play) (see Fig. 1). Based on previous reports^10,41^, we adapted the double Still-Face Paradigm to include smartphone disruptions during the Still-Face conditions. Mothers were instructed that during 10 min of interaction with their infant, they would be asked to interrupt their play for 2 times, each for a duration of 2 min. During these interruptions, the mothers were instructed to look at their phone, have no (verbal or non-verbal) interaction with their infant and be (emotionally) unresponsive. During the periods of play (free play; FP1 and FP2) and reunion, the mother played with her child with the instructions “*play with your child as you usually do at home*”. The mothers were provided with a similar basket of toys that made minimal sound (e.g., plastic book, stuffed animal). Note here that the free play and reunion periods are the same in terms of condition/task. However, the mother knows that in the final 2 min she can reunite with her infant and no more disruptions will come, which makes the reunion period qualitatively different than the free play periods. Our previous analysis using the behavioral data from this sample confirmed that our smartphone-adapted SFP elicited a clear behavioral “still-face effect” in the infants, with lower positive affect and higher negative affect during the still-face conditions^47^.

### Dual-EEG measurement

**Fig. 4:**
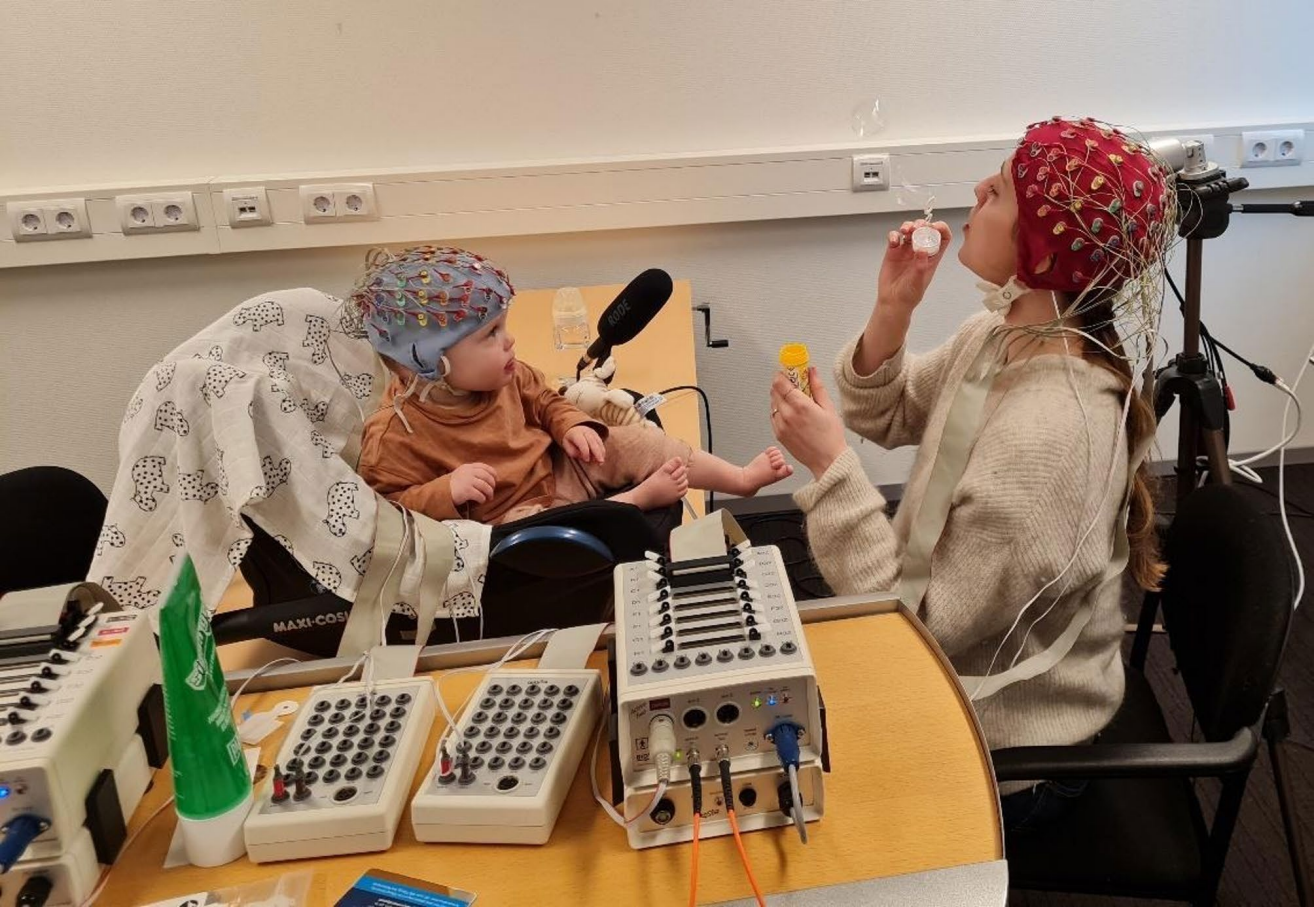
Mother-infant dual-EEG setup using biosemi ActiveTwo. The baby is seated in a baby car seat placed on the table in front of their mother. Note that the task displayed in the photo was not part of the Still Face Paradigm, but of the Bubbles task (no photos were taken during the SFP). Photo was used with consent of the mother depicted in the photo.

The dual-EEG measurements were recorded using two BioSemi Active Two systems that were interconnected, with each system consisting of 64 channels. Electrodes were positioned according to the 10–20 system, and the sampling rate was adjusted to 512 Hz. During the measurement, mothers sat in a chair facing their infants. The infants were placed in a car seat on a (low) table (see Fig. 4 for an example of the set-up). Our hyperscanning EEG set-up, and those of other labs, has been described in detail in Barraza, et al.^68^.

### EEG preprocessing

Before analysis, data preprocessing was conducted using Brain Vision Analyzer software (Brain Products). The preprocessing steps included high-pass filtering at 1 Hz, low-pass filtering at 30 Hz, and the application of a 50 Hz notch filter to remove line noise. Additional preprocessing involved exclusion of channels with poor signal quality, and, if necessary, interpolation of missing data considering neighboring channels (up to 10% of channels). The data was then segmented into 2 min epochs consistent with the fives phases of the SFP, resulting in 5 files per dyad. The data was then further segmented in 2 s epochs with 50% overlap. Prior studies have suggested that each epoch should encompass a minimum of three cycles of the lowest frequency under examination and should be long enough to minimize the signal-to-noise ratio^69,70^. At the same time, increasing the number of epochs improves the detection of phase-locked activity^69^. Therefore, given that the lowest frequency considered in the analysis was 3 Hz, the selection of 2-second epochs was deemed an optimal choice for our analysis. Here, the 2 s epochs were hyper-epochs, consisting of time-locked EEG data from both mother and infant. Following the rejection of artifacts, the average number of retained hyper-epochs for the Free Play 1, Still-Face 1, Free Play 2, Still-Face 2, and Reunion conditions were 102.125 (SD = 18.43), 101.48 (SD = 23.76), 97.63 (SD = 22.2), 101.06 (SD = 26.97), and 98.29 (SD = 20.21), respectively.

### Brain-to-brain synchrony analysis

To reduce the number of comparisons and address the problem of volume conduction between neighboring electrodes, the present analysis of mother-infant brain-to-brain synchrony was restricted to a total of 12 EEG channels. By limiting the number of channels, the study aimed to improve the signal specificity and reduce the potential confounding effects of neighboring electrodes, thereby enhancing the accuracy and interpretability of the results. The channels were located in the following regions, from anterior to posterior: mid-frontal (F3, F4), frontal (F7, F8), central (C3, C4), temporal (T7, T8), parietal (P3, P4), and occipital-temporal (P7, P8) regions. Prior research has reported that these regions show significant inter-brain synchrony during ecologically valid experiments e.g.,^33,36,52,71^.

The current study analyzed EEG data from each condition using the weighted Phase Lag Index (wPLI) to detect brain synchrony between a mother and her infant. The wPLI was proposed by Vinck, et al.^44^as an improved version of the PLI and has been increasingly used by previous mother-infant dual-EEG studies e.g.,^33,36,71^. The wPLI was found to be more sensitive in detecting phase synchronization, while being less sensitive to additional, uncorrelated noise sources^44^. The analysis was carried out using the HyPyP library in Python^72^.

After band-pass filtering in the specific frequency bands of interest, which were the infant theta (3–5 Hz) and alpha (6–9 Hz)^73^, the Hilbert transform was used to estimate instantaneous phase angles for each frequency. These phase angles were then utilized to determine wPLIs for each epoch between the mother’s and infant’s channels.

To analyze the interaction between the two brains in each condition, the trial-averaged wPLI was calculated for each combination of the selected 12 electrodes located on the two EEG caps. Specifically, for each pair of electrodes *j* and *k*, where j corresponds to an electrode on the baby’s EEG cap and *k* to an electrode on the mother’s EEG cap, the wPLI was computed. The wPLI was estimated using the adjusted formula of Vinck, et al.^44^.

$$wPLI_{N} = \frac{{\frac{1}{N}\sum\limits_{{i = 1}}^{N} {\left( {\left| {I\left( {\Delta \phi _{i} } \right)} \right| \cdot sign\left( {I\left( {\Delta \phi _{i} } \right)} \right)} \right)} }}{{\frac{1}{N}\sum\limits_{{i = 1}}^{N} {\left| {I\left( {\Delta \phi _{i} } \right)} \right|} }}$$

- *N*: The number of trials,
- $\:I\left(\varDelta\:{\varphi\:}_{i}\right)$: The imaginary part of the phase difference between the two signals for the -trial,
- sign: The sign of the imaginary part of the phase difference for the -th trial. It gives the direction of the phase lead/lag between the two signals.

The wPLI ranges from 0 to 1. The value of 0 indicates that the two signals do not show any consistent phase difference. The values > 0 signify that there is a consistent phase relationship between the two signals, meaning that one signal tends to either lead or lag the other. The higher the value, the stronger and more consistent this phase difference is. A value of 1 indicates perfect synchronization in phase, where one signal consistently leads or lags the other throughout the entire period with no phase reversals or inconsistencies (MNE-Python Developers, n.d.).

Before computing global inter-brain synchrony, which was the dependent variable in this study, a statistical test based on surrogate data was performed as described by^74^ and adapted to the inter-brain synchrony analysis to differentiate real synchrony from background fluctuations. The following steps were taken to determine the presence of significant synchrony between the mother and infant’s EEG in each 2 s hyper-epoch:

1. wPLI was computed between the EEG signals of the mother and infant for a given trial.
2. The phase angles from the Hilbert transform were shuffled.
3. wPLI was computed between the shuffled EEG signals of the mother and infant.
4. Steps 2–3 were repeated 200 times.
5. A statistical test was conducted by comparing a single wPLI obtained from the original EEG data with 200 wPLIs obtained from randomized EEG data. A significance level of 5% was employed to assess whether the observed wPLI value significantly deviated from the wPLI values obtained through randomization.

If the observed wPLI was higher than 95% of the randomized wPLIs, it was concluded that the observed synchrony was not due to random noise but rather due to the true synchronization between the EEG of the mother and infant. The trials in which wPLIs were found insignificant (i.e., the observed wPLI value was lower than more than 5% of the randomized wPLI), were not included in the computations of the global inter-brain synchrony.

To quantify the global brain-to-brain synchrony between mother and infant, the average of all the pairwise wPLIs obtained for each frequency band and condition was computed. This resulted in a total of ten wPLI mean values per subject, representing the mean wPLI for each condition (five conditions of the SFP) and frequency band (theta or alpha).

Notably, we also conducted our analyses on using Phase Locking Values (PLV), since PLV values have been used in several other studies on brain-to-brain synchrony^45,75^. When performing the validation analyses (step 5), in which each epoch was compared to a surrogate dataset of randomized signals, we found that over 77% of the epoch had to be excluded. This indicates that phase synchrony in 77% of randomized epochs was not significantly different from the original EEG signals. For the wPLI values, less than 5% of the epochs had to be excluded.

### Statistical analysis

All analyses were performed in SPSS version 27 (IBM Statistics). First, descriptive analyses were performed to compute means and standard deviations of the main variables in our sample. We then conducted a series of Two-Sampled t-tests to explore differences in global brain-to-brain synchrony between mother-son and mother-daughter dyads. We also ran Pearson’s correlations to check associations between the global brain-to-brain synchrony values and infant’s age. The brain-to-brain metrics were subsequently checked for normality. Since most metrics were not normally distributed, we proceeded to run non-parametric tests. First, a Friedman test was used to assess whether there was a significant difference between the means of the five conditions of the Still-Face Paradigm. If significant, the Friedman test was followed-up by post hoc testing using Pairwise Wilcoxon Signed-rank tests. We used FDR correction to control for multiple testing. Global wPLI values per condition of the SFP were used as dependent variables. Both sex and age of the infant were added to the model as covariates.

Next, we performed topographical analyses to examine which inter-brain regions showed significant differences between the Still-Face and Free Play/Reunion conditions. To perform this analysis, we first grouped the electrodes into 5 regions to reduce the number analyses: frontal (F8, F7), central-parietal (C4, C3), occipital (P8, P7), left temporal (T7) and right temporal (T8). We then ran a series of paired-sample t-tests with a one-sided hypothesis (‘less’) to compare trial-averaged values of two conditions: the combined Still-Face conditions (SF1, SF2) with the combined conditions (FP1, FP2, RU) across the specified brain regions of the baby and the mother. The t-tests were performed for each inter-brain region-combination of 5 regions of the mother with 5 regions of the infant separately per frequency band (theta and alpha), resulting in 50 total tests. False Discovery Rate (FDR) correction^76^ was used to control for multiple testing.

## Data Availability

Computed brain-to-brain synchrony data that support the findings of this study have been deposited as an SPSS datafile on Open Science Framework (OSF) and can be retrieved via https://osf.io/rsy84/. When using this data, please cite our work accordingly, including the OSF page for the Brains in Sync Study. Reference: van den Heuvel, M.I., Mosińska, A., Turk, E. & Alimardani, M. (2025, June 3). Brains in Sync Study. https://doi.org/10.17605/OSF.IO/KRDN5.
